# Synaptic plasticity and cognitive impairment consequences to acute kidney injury: Protective role of ellagic acid

**DOI:** 10.22038/IJBMS.2022.62015.13729

**Published:** 2022-05

**Authors:** Alireza Sarkaki, Khojasteh Hoseinynejad, Maryam Khombi Shooshtari, Mohammad Rashno

**Affiliations:** 1 Department of Physiology, Faculty of Medicine, Persian Gulf Physiology Research Center, Medical Basic Sciences Research Institute, Ahvaz Jundishapur University of Medical Sciences, Ahvaz, Iran; 2 Chronic Renal Failure Research Center, Ahvaz Jundishapur University of Medical Sciences, Ahvaz, Iran; 3 Persian Gulf Physiology Research Center, Medical Basic Sciences Research Institute, Ahvaz Jundishapur University of Medical Sciences, Ahvaz, Iran; 4 Cellular and Molecular Research Center, Medical Basic Sciences Research Institute, Ahvaz Jundishapur University of Medical Sciences, Ahvaz, Iran; 5 Department of Immunology, Faculty of Medicine, Ahvaz Jundishapur University of Medical Sciences, Ahvaz, Iran

**Keywords:** AKI, Ellagic acid, Memory, Rat, Synaptic plasticity

## Abstract

**Objective(s):**

The goal of the current experiment was to define the efficacy and underlying molecular mechanisms of Ellagic acid (EA) on acute kidney injury (AKI) induced impairment in cognitive and synaptic plasticity in rats.

**Materials and Methods:**

Administration of 8 ml/kg glycerol (intramuscular) was used to establish the AKI model. Injured animals were treated by EA (25, 50, and 100 mg/kg, daily, gavage) for 14 consecutive days. To approve the renal injuries and the effects of EA on AKI, creatinine values in serum and urea nitrogen (BUN) values in blood were measured. Cognitive performance was investigated using the Morris water maze test. In vivo long-term potentiation (LTP) was recorded from the hippocampus. Then, the level of IL-10β and TNF-α levels were measured using ELISA kits. The integrity index of the Blood-brain barrier (BBB) was assessed by extravasation of Evans blue dye into the brain.

**Results:**

Glycerol injection increased blood urea nitrogen (BUN) and serum creatinine (Scr) levels significantly in the AKI group, and EA treatment resulted in a significant reduction in BUN levels in all concentration groups. Also, a significant reduction in the cerebral EBD concentrations was demonstrated in EA treatment rats. Moreover, the indexes of brain electrophysiology, spatial learning, and memory were improved in the EA administrated group compared with the AKI rats.

**Conclusion:**

The current experiment demonstrated the efficacy of EA in hippocampal complication and cognitive dysfunction secondary to AKI via alleviating the inflammation.

## Introduction

Acute kidney injury (AKI) which is prominent as acute renal failure, is a progressive disorder described by rapid assemblage of end components of nitrogen metabolism, such as serum urea and creatinine, or decreased urine outflow, caused by reduction of the kidney’s excretory capacity (1-4).

Rhabdomyolysis (RM) is a clinical syndrome that is defined as a massive breakdown of skeletal muscles to the damaging intracellular contents which releases in the systemic circulation. In RM, the components of muscle fibers such as myoglobin (mg), aspartate aminotransferase, creatine phosphokinase, alanine aminotransferase, and lactate dehydrogenase may leak into the systemic blood circulation (5-7). The major complications of the rhabdomyolysis condition are AKI. In this context, many experiments have focused on the factor which can affect this destructive process. The animal models of renal injury induced by a single intramuscular injection of glycerol in rats is a well-known method to identify the underlying cellular and molecular mechanisms in process of AKI as a secondary complication of RM (5). 

In the *in vivo* model of AKI which was established using glycerol, the myoglobin heme caused increased lipid peroxidation in proximal tubules. Furthermore, myoglobin heme stimulates the production of inflammatory mediators, containing cytokines and chemokines, which can activate leukocytes, resulting in tubular necrosis in the kidney cortex (3). During the kidney injury process, the brain might have interacted through intensification of damage induced by inflammatory factors, excerebration of leukocytes, oxidative damage, and dysregulation of membrane channels. Accordingly, episodes of AKI can lead to subsequent progressive kidney and brain injuries (4). AKI could accompany various brain and hippocampal dysfunctions due to alteration of permeability in the blood-brain barrier. The hippocampus dysfunction as the main structure involved in learning and memory is very sensitive to systemic inflammations like renal injuries, and can finally lead to cognitive impairment in AKI patients (1, 8, 9). Exacerbation in free radicals and cytokine factors in the brain tissue secondary to AKI has been reported to correlate with neuronal cytotoxicity and apoptosis. Systemic free radical accumulation with BBB impairment can lead to expansion of free radicals in various parts of the brain like the hippocampus (8).

 Ellagic acid (EA), a natural polyphenolic compound is found in most soft and hard-shelled fruits and plant extracts (10-12). Studies, *in vitro* and *in vivo,* suggested that EA has antioxidant, antibacterial, anti-inflammatory, and anti-cancer properties (13, 14). Furthermore, EA was reported as neuroprotective and improved learning and memory in neurodegenerative disorders in rats (12, 13, 15-17). In view of its potential therapeutic value, it is reported that EA has a protective effect against cisplatin-induced nephrotoxicity (18), kidney protective effect against Carbone tetrachloride (CCl4) induced oxidative damage and inflammation (19), renal protective effect in diabetic rats (20), and protective effect against nicotine-induced oxidative damage and apoptotic changes in rat kidney (21). However, the molecular pathway of AKI-induced brain malfunction and its mechanism basis are poorly understood (22).

Therefore, in this study, we examined the memory performance, synaptic plasticity of hippocampal neurons, microvascular permeability, and biochemical changes in the brain after 14 days of treatment with different doses of EA in an animal model of AKI induced by glycerol in rats.

## Materials and Methods

***Chemicals and drugs***

Glycerol and EA were procured from Sigma-Aldrich Chemical Co. (St. Louis, MO, USA). The chemicals and reagents which are not mentioned in the present experiment were of analytical grade.

***Animal care and ethics***

Sixty adult healthy male rats (Wistar, Age: approximately 3 months and weight: 200–250 g) were housed at controlled temperature (22 ± 2 °C) under a 12-hr light/dark cycle with access to enough food and water. All procedures were done considering the Ethics of Experimental Animal Committee (Ahvaz Jundishapur University of Medical Sciences, Iran) and in accordance with the principles outlined in the NIH Guide for the Care and Use of Laboratory Animals (with ethics code IR.AJUMS.ABHC.REC.1399.051).

***Experimental design***

All groups of rats were maintained in standard conditions for 1 week and then deprived of water for 24 hr before the beginning of the study. The animals were divided into five equal groups. 1) Control; the animals which received oral administration of DMSO 5% in saline as a vehicle for two consecutive weeks. 2) AKI; rats received ½ of glycerol concentration (8 ml per kg) using an intramuscular manner (23) and, received an oral administration of DMSO 5% in saline as a vehicle for two consecutive weeks after glycerol administration. 3-5) Treated groups, AKI +EA1, EA2, and EA3, rats in these groups received different doses of EA (25, 50, and 100 mg/kg/day orally)for two consecutive weeks after glycerol administration.

***Assessment of kidney function***

At the end of the experiments, all rats were sacrificed under deep and irreversible anesthetized with sodium thiopental (neuronal, 80 mg/kg, IP). Blood samples were collected from the heart, then allowed to clot and centrifuged to obtain serum. The biochemical parameters of blood urea nitrogen (BUN) and creatinine (Cr) levels were measured in serum using the standard diagnostic kits (Span Diagnostics, Gujarat, India).

***Assessment of the permeability in the blood-brain barrier***

For this purpose, the BBB integrity was assessed via the Evans blue dye (EBD) method. Briefly, five animals from each group were anesthetized and 20 mg/kg of EBD solution (2% Evans blue in normal saline, Sigma, Germany) was injected through the tail vein of the rat. Sixty min later, the thoracic cavity was exposed in anesthetized rats and then perfused with 300 ml of normal saline. This procedure was done through the left ventricle until exiting colorless fluid to eliminate the intravascular EBD. After this step, the brain tissue was collected, weighed, and homogenized using trichloroacetic acid. The collected tissues were incubated freeze (4 °C) for 3 min. Then, the collected supernatant was centrifuged (4000 rpm for 30 min). Finally, the concentration of EBD was assessed by reading the absorbance (620 nm) using the spectrophotometry (Biochrome, Cambridge, UK) method.

The level of dye within brain tissue increased considering increasing vascular permeability and enhancement of blood-brain barrier dysfunction (22, 24, 25).

***Evaluation of Morris water maze (MWM) indexes***

Spatial memory function was evaluated using MWM examination which included 4 consecutive acquisition sessions and memory retention or Day 5 probe trial which was done 24 hr after the final session. The MWM examination apparatus included a circular black-painted metal pool (diameter: 150 cm and height: 60 cm) which was filled with 25 ± 1 °C water (Depth: 40 cm). The apparatus was located inside a room and surrounded by various spatial cues. The pools were divided into North, South, West, and East quadrants. The animal’s behavior including swim speed, latency, navigation path, and path length were monitored and followed via a video camera, which was positioned directly above the center of the pool. Firstly, the rats learned to identify the platform which was invisibly submerged (diameter: 12 cm and 2 cm underwater surface) and fixed in one of the four quadrants for four 60-sec trials per day. In each experiment, all rats were released randomly from one of the four pool quadrants and remained to find and jump on the platform between each trial for 30 sec. Latency to reach the escape platform was measured as acquisition. In the probe test, 24 hr after the acquisition session (on the 5th day), the platform was removed and the rats were allowed to swim for one 60 sec probe trial to assess memory retention. The escape latency index in the time of spatial learning experiment and also the time during the target quadrant test in percentage were recorded by Ethovision software (ver. 7). The swimming speed was evaluated in all rats to identify the differences in motor ability conditions during swimming (26, 27).

***Electrophysiological studies***

Electrical activity of the dentate gyrus (DG) area of the brain hippocampus was recorded in anesthetized rats (ketamine (90 mg/kg) mixed with xylazine (10 mg/kg)) at the end of the Morris water maze test. In order to record the field potentials as long-term potential (LTP), the animal’s head was fixed in a stereotaxic apparatus and the skull surface was exposed for implantation of the microelectrodes. The non-electric heating pad was applied to keep normal body temperature (36.5±0.5 °C). One stimulating stainless steel microelectrode (bipolar) was placed into the left perforant path. Also, another tungsten recording microelectrode was implanted into the hippocampal (ipsilaterally) with coordinates of the DG area according to the atlas of Paxinos and Watson, respectively(28). In order to decrease traumatic injuries to the brain tissues, the mentioned microelectrodes were lowered gradually from dura to the PP and were positioned in the high fEPSP area (29).

***LTP induction***

The dentate gyrus field potentials were evoked using stimulation of the PP area. At the beginning of the experiments, the test stimulation intensity was applied to elicit afEPSP and recorded at eight various currents intensities (50, 100, 150, 200, 250, 300, 350, and 400 µA). After recording the baseline trace, a high-frequency stimulation procedure was performed to induction of LTP(29). The severity of the HFS stimulus was measured as the evoked slope value of fEPSP and amplitude of population spikes (PSs) in 80% of the maximum value of responses. The voltage differences among the negative deflection (firstly) and a positive wave were measured and reported as the PS amplitude post-tetanic stimulation. Moreover, the fEPSP slope value was documented as the maximum slope among the fEPSP (initial point) and the wave (first positive peak) in order to assess synaptic efficacy. The amplification of the extracellular field potentials was done in ×100 with 0.1 Hz–3 kHz filtration (12, 30).

***ELISA assay on collected tissues***

In order to determine the cytokines in the hippocampus, 5 rats from each group were deeply anesthetized and perfuse through the heart using normal saline. After this step, the brain tissues were collected, and the hippocampi area was dissected on ice, washed with saline, and then preserved in a freezer (-80 °C) for the consequence of ELISA analysis. 100 mg hippocampi tissues were homogenized using cold PBS plus protease inhibitor cocktail and centrifuged (10000×g, 4 °C) for 20 min. Then, the supernatant samples were collected and the protein concentration was assessed using the Bradford method. Also, TNF-α and IL-10 levels were detected via ELISA kits and reported as picograms per milligrams of protein (pg/mg) (31-33).

***Statistical analysis***

All data were shown as mean ± SEM. The data (MWM test and LTP examinations) was assessed by repeated measure two-way ANOVA followed by Tukey’s *post-hoc* test. One-way ANOVA and Tukey’s *post hoc* test were used for analyzing other data. Values of *P*<0.05 were considered to be statistically significant. Statistical analyses were done using GraphPad Prism 6 software.

**Figure 1:**
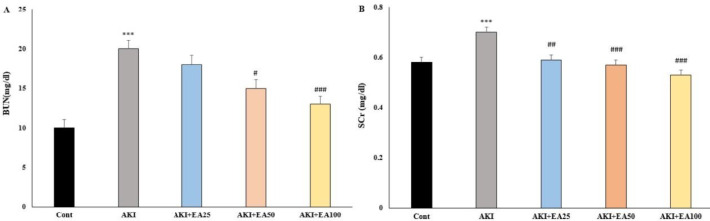
Blood urea nitroge n (BUN; mg/dL; (A)) and seru m creatinine (Scr; mg/dl; (B)) in all groups, including: Cont: control, AKI: acute kidney injury, EA: ellagic acid

Results are demonstrated as mean ± SEM. ***P<0.001 versus the control group;#*P*<0.05, ##*P*<0.01, and ###*P*<0.001 versus the AKI group

**Figure 2:**
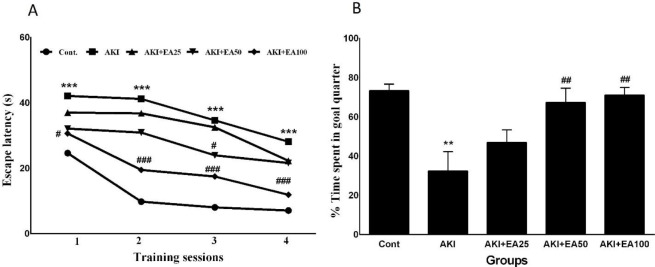
Blood Brain Barrier permeability in AKI mode in all groups, including: Cont: control, AKI: acute kidney injury, EA: ellagic acid

Results are demonstrated as Mean ± SEM. ***P<0.001 versus the control group; ##*P*<0.01 versus the AKI group

**Figure 3:**
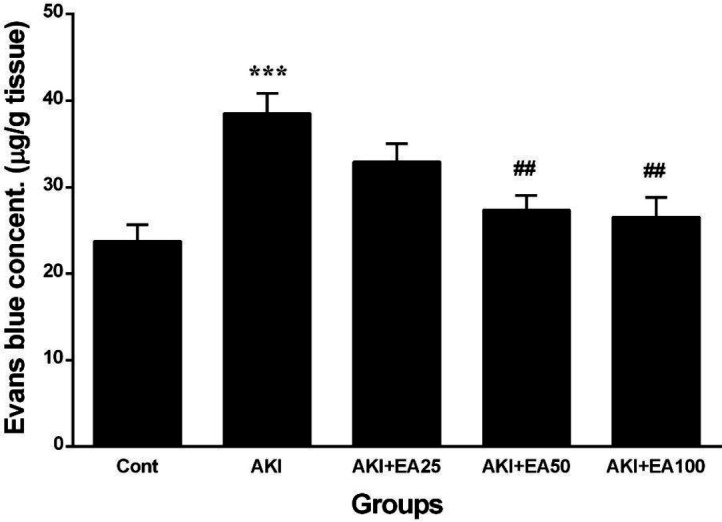
Memory performance in the AKI model in all groups, including: Acquisition trials (for 4 days), with hidden platform training, followed by a probe trial. (A) Escape latency in the acquisition trial. (B) Percentage of time spent in the target quadrant during the probe trials Cont: control, AKI: acute kidney injury, EA: ellagic acid

Results were expressed as Mean ± SEM. ***P<0.001 versus the control group; #*P*<0.05, ###*P*<0.001 versus the AKI group

**Figure 4:**
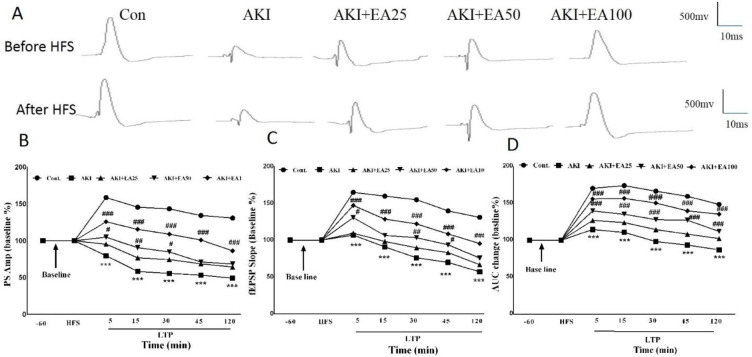
Synaptic plasticity in AKI rats; A) Recorded sample traces. B) Population spikes (PS) amplitude (5, 15, 30, 45, and 120 min after the HFS). C) fEPSP slope (5, 15, 30, 45, and 120 min after the HFS). D) Area under curve (AUC) for PSs during 5, 15, 30, 45, and 120 min after the High-frequency deep brain stimulation (HFS) Cont: control, AKI: acute kidney injury, EA: ellagic acid

Results were expressed as Mean ± SEM. **P<0.01 and ***P<0.001 versus the control group; #*P*<0.05 and ##*P*<0.01 versus the AKI group

**Figure 5:**
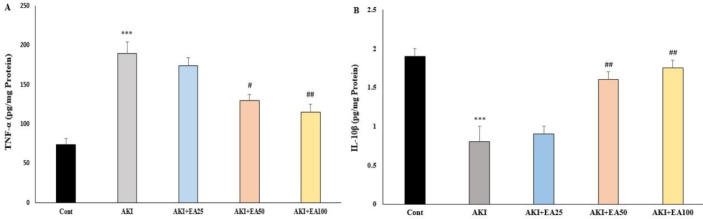
Inflammatory parameters (TNF-α, IL-10) in the hippocampus in AKI rats. A) Hippocampal TNF-α content B) Hippocampal IL-10β content Cont: control, AKI: acute kidney injury, EA: ellagic acid

Results were expressed as Mean ± SEM. ***P<0.001 versus the control group; #*P*<0.05 and ##*P*<0.01 versus the AKI group

## Results

***Biochemical analysis***

Glycerol caused renal function reduction, as shown by high concentration levels of creatinine and urea in the serum. The effects of EA on kidney function in rats are shown in Figure 1. Glycerol injection in the AKI group significantly increased serum creatinine (Scr) and blood urea nitrogen (BUN) concentration compared with the control rats (*P*<0.001; Figure 1A, B). EA treatment resulted in a significant reduction in BUN levels in AKI+EA50 and AKI+EA100 groups in comparison to the rats model of AKI, respectively (*P*<0.05, *P*<0.001; Figure 1A, F 4,20 = 13.52). Also, EA treatment resulted in a significant reduction in Scr level in AKI+ EA25, AKI+EA50, and AKI+EA100 groups compared with the AKI group, respectively (*P*<0.01, *P*<0.001, and *P*<0.001; Figure 1B, F 4,20 = 14.08).

***BBB Permeability***

The brain tissue content of EBD as an index for BBB integrity assessment is shown in Figure 2. Glycerol injection significantly increased the cerebral EBD concentrations in the AKI group compared with the control group (F4, 20= 8.039, *P*<0.01). EA treatment resulted in a significant reduction in the cerebral EBD concentrations in AKI+EA50 and AKI+ EA100 groups compared with the AKI group (*P*<0.01). 

***Spatial learning and memory***

As the results show in Figure 3A, the latency time in the MWM task was diminished in all experimental groups during four days of acquisition sessions which demonstrated that all rats learned to identify the hidden platform. The analysis result of the latency time in finding the hidden platform demonstrated effective properties of EA treatment (F4,24=41.28; *P*<0.001) and days (F3,18=29.73; *P*<0.001). However, the results showed that there were no remarkable effects of days×treatment interaction (F12,72=0.172; *P*> 0.05) on any training day during the study period. Also, the data showed glycerol injection causes prolongation of latency in finding the hidden platform in AKI rats compared with the control group on days 1, 2, 3, and 4 of the training period (*P*<0.001); while EA treatment for two continuous weeks reveals a remarkable diminish in the escape latency measurement in the AKI+EA50 (*P*<0.05) and AKI+EA100 groups (*P*<0.05 and *P*<0.001) compared with the AKI group. In the probe-trial test, the time spent (percentage) in the objective quadrant was considered in the assessment of memory in trained rats. As shown in Figure 3B, the AKI rats spent a short time in the target quadrant compared with the control (F4,30=7.296, *P*<0.001); However, EA treatment in AKI+EA50 and AKI+EA100 groups significantly enhanced the percentage of time spent in the objective quadrant compared with the AKI group (*P*<0.01).

***Hippocampus electrical activity***

The sample traces were obtained from the DG area in the hippocampal region pre and after 400 Hz stimulation as shown in Figure 4A. The results of PS amplitude measurement demonstrate remarkable effects of EA-treatment (F4,16=9.115; *P*<0.001) and time (F6,168=41.82; *P*<0.001) (Figure 4B). Moreover, there were remarkable effects of time×treatment interaction (F24,96=6.404; *P*<0.001) for PS amplitude recording at various times. Also, glycerol injection significantly decreased PS amplitude in all LTP recording times in the AKI group compared with the control group (*P*<0.001). Whereas, EA treatment for 14 days significantly enhanced the PS amplitude in the AKI+EA50 (*P*<0.05 and *P*<0.01) and AKI+EA100 (*P*<0.001) groups compared with the AKI group. According to Figure 4C, the results of analysis of the slope values for fEPSP reveal the remarkable effects of treatment with EA (F4,16=14.65; *P*<0.001) and time (F6,24=12.60; *P*<0.001). Furthermore, there were remarkable properties in time×treatment interaction (F24,96=5.925; *P*<0.001) in fEPSP slope in various times. Moreover, the fEPSP slope showed remarkable decreases in the AKI group compared with the control group (*P*<0.001), while EA treatment for two consecutive weeks significantly increased the fEPSP slope in the AKI+EA50 (*P*<0.05 and *P*<0.01) and AKI+EA100 groups (*P*<0.001) compared with the AKI group. As shown in Figure 4D, the results showed significant effects of treatment with EA on area under curve (AUC) of PS (F4,16=13.40; *P*<0.001) and time (F6,24=56.38; *P*<0.001). Also, there were significant effects of time×treatment interaction (F24,96=8.261; *P*<0.001) for the AUC of PS at various times. Data showed that the effects of glycerol injection on the AUC of PS were significantly decreased in the AKI group compared with the control group (*P*<0.001); while EA treatment for two weeks in a row showed remarkable enhancement in the AUC in the AKI+EA50 (*P*<0.001) and AKI+EA100 groups (*P*<0.001) in comparison with the AKI rats.

***Hippocampal levels of inflammatory mediators***

Figure 5A reveals the effects of EA treatment (two consecutive weeks) on the hippocampal levels of TNF-α after glycerol injection-induced AKI (F4,20=9.254; *P*<0.001). These data showed that glycerol injection significantly increased hippocampal level of TNF-α in the AKI group compared with the control (*P*<0.001), while, EA treatment in AKI+EA50 and AKI+EA100 groups decreased hippocampal levels of TNF-α compared with the AKI group (*P*<0.05 and *P*<0.01). As shown in Figure 5B glycerol injection significantly(F4, 20=11.66; *P*<0.001)decreased hippocampal levels of IL-10 in the AKI group compared with the control group. But treatment of AKI rats with EA significantly(*P*<0.01 and *P*<0.01) increased levels of IL-10 in the AKI+EA50 and AKI+EA100 groups compared with the AKI group.

## Discussion

The current experiment documented that AKI rats had remarkable hippocampal long-term potentiating deficits and memory impairments, which were assessed by electrophysiological and Morris water maze methods. Moreover, BBB permeability as a physiological impairment index was changed in the AKI rats. This study reported that treatment with EA could improve these behavioral and physiological dysfunctions, which may be related to the anti-inflammatory properties of EA. Rhabdomyolysis (RM) is defined as the breakdown of skeletal muscles which is caused by outflow of muscle enzymes and release of toxic compounds into the blood circulation leading to AKI (3, 34, 35). These compounds are filtered out via glomeruli and increase the risk of development of AKI which might lead to enchaned rate of mortality worldwide. This mal-condition could relate to the extra-renal complications which occur secondary to distant-organ involvement with a special and distinct profile of injurious mediators (36-38). After AKI, neurologic complications are major causes of mortality (39). Accordingly, using safe agents to prevent, treat, or minimize AKI conditions is essential. This experiment was carried out to identify the potential efficacy of EA against cognitive impairment after the RM-induced AKI model in rats via administration of glycerol. The obtained data demonstrate an increase in indexes of renal function, such as Scr and BUN. The increased Scr and BuN levels show the critical role of renal injury markers (6, 40). This study showed that EA treatment ameliorated the alterations in kidney function parameters in RM-induced AKI by decreasing Scr and BUN. EA, a natural polyphenolic compound, exhibits various pharmacological and biological activities. However, several studies documented that administration of EA provides remarkable protection against cisplatin-induced nephrotoxicity by decreasing the plasma and urea creatinine levels (18, 41, 42). *In vivo* AKI models have shown that the inflammation process is related to intensifying the adverse effects on remote organs such as the brain (43). Increased levels of inflammatory factors have been shown in the hippocampus tissue during the AKI process (9). Therefore, we found that AKI would also lead to brain inflammatory changes. In this study, TNF-α was elevated in the hippocampus in the glycerol group. In addition, glycerol injection caused a decrease in IL-10β levels in the hippocampus. In the treatment group with EA in a concentration-dependent manner, the levels of TNF-α in hippocampal tissue significantly dropped and concentrations of IL-10β increased; which may cause improvement of the cognitive condition and hippocampal LTP in response to the BBB repairmen. These results clearly demonstrated that EA, probably via its anti-inflammatory properties, ameliorates the consequences of glycerol-induced AKI. In the brain, several studies on the blood-brain barrier (BBB) documented an important role in the homeostasis of the brain (44). The BBB is defined as a defensive physical and metabolic barrier between the CNS and peripheral circulation that helps to preserve the microenvironment of the brain areas. The structure of BBB is made up of endothelial cells (non-fenestrated) with tight junctions that are responsible for decreases in permeability of the barrier (45, 46). It is now believed that disruption of the BBB is the major episode of brain injury after AKI (4, 9). In response to the AKI, inflammation will occur due to endothelial dysfunction, oxidative stress, and other multiple risk factors (4). Subsequent to inflammation, release of cytokines occurs through activation of inflammatory cells, astrocytes, microglia, and BBB endothelium (47, 48). The chemotactic role of cytokines could lead to alteration in the construction of the endothelial tight junctions, which are vital elements of the BBB (49). Cytokines also activate microglial cells and donate to dysregulation of water channels (4). An increase in BBB permeability leads to changed CNS microenvironment, causing CNS dysfunction (50). Accordingly, in the current experiment, we assessed brain microvascular function by Evans’s blue dye extravasations method and showed that AKI leads to enhancement of brain microvascular permeability. Our data are in line with the studies which documented BBB derangement in both acute and chronic uremic encephalopathy conditions (9). However, the data of the current animal experiment showed that the BBB function was improved in EA-administrated animals which may mediate by properties of EA against BBB disruption through inhibition of inflammatory pathways. Mashhadizadeh *et al*. indicated disrupted BBB permeability after TBI was improved with EA(13). Although, numerous studies have documented various properties of EA, including neuroprotective effects, anti-inflammatory properties, and anti-oxidant and free-radical scavenging effects (51, 52). The main target mechanism by which EA improved these effects has not been fully identified and seems to need more investigation. As seen in the AKI group in the present study it was demonstrated that the hippocampal may be involved in the AKI process as a critical region responsible for learning and memory which may lead to cognitive and synaptic plasticity dysfunction. However, we reported that the function of memory was significantly reduced in AKI rats which showed MWM performance. This data supports other studies on memory loss due to AKI in animal models (9, 53, 54). Furthermore, we showed that EA treatment promotes brain plasticity and prevents memory loss; which is related to the brain’s hippocampal functions. LTP is a molecular mechanism in memory conditions and also is a long-lasting increase in synaptic plasticity; much focus on LTP has been in the hippocampus neural circuits (55-57). The hippocampus LTP is a well-documented bio-index for assessing learning and memory function. Our electrophysiological study reported that administration of EA could cause enhancement of PS amplitude, fEPSP slope, and AUC following HFS in the AKI rats. These data showed that EA-treatment leads to synaptic plasticity enhancement through the DG area of the hippocampus with pyramidal neurons. An earlier study showed that EA attenuated synaptic plasticity impairment in traumatic brain injury models in rats (13). It has been reported that an enhanced level of TNF-α leads to inhibition of LTP induction in the CA1 and DG region in rat hippocampus to the pathophysiological values (58, 59). Surprisingly, it has been reported that inflammation in peripheral organs as shown in kidney tissue can affect hippocampal synaptic transmission (60). It is suggested that parallel changes in aminomethylphosphonic acid sub-units and NMDA receptors such as GluR and NMDA subtype 2B receptors are involved in this destructive process. Glutamate release enhancement associated with down-regulation of cannabinoid receptors (61), can potentiate release of transmitter secondary to systemic inflammation. Increases in bio-synthesis of systemic pro-inflammatory cytokines can stimulate BBB receptors on sensory afferents or within the circumventricular organs, which mediates the inflammatory cascade in the brain(8). AKI could affect the hippocampus tissue as a major area of cellular inflammation due to synthesis of soluble inflammatory components and destruction of BBB. Disruption of BBB permeability secondary to AKI allows cytokines to penetrate the brain, which leads to edema and inflammation. Moreover, activation of microglia in response to cytokines could increase the process of BBB disruption and brain dysfunction (62), while EA treatment has the potential to ameliorate these changes via its anti-inflammatory efficacy. According to the mentioned results, our study presented pieces of evidence on EA advantages to consider as a useful strategy to diminish the progression of AKI.

## Conclusion

Treatment with EA showed a potential dose-dependent neuroprotective action against AKI- induced cognitive impairment and hippocampal electrophysiological deficit, especially a dose of 100 mg/kg which ameliorated the investigated parameters. The ability of EA to suppress inflammatory mediators makes it a potential candidate for inhibition of AKI progression.
